# Molecular Hydrogen Increases Quantitative and Qualitative Traits of Rice Grain in Field Trials

**DOI:** 10.3390/plants10112331

**Published:** 2021-10-28

**Authors:** Pengfei Cheng, Jun Wang, Zhushan Zhao, Lingshuai Kong, Wang Lou, Tong Zhang, Dedao Jing, Julong Yu, Zhaolin Shu, Liqin Huang, Wenjiao Zhu, Qing Yang, Wenbiao Shen

**Affiliations:** 1 Laboratory Center of Life Sciences, College of Life Sciences, Nanjing Agricultural University, Nanjing 210095, China; 2020216037@njau.edu.cn (P.C.); 2020116099@stu.njau.edu.cn (J.W.); 2021816131@stu.njau.edu.cn (Z.Z.); 2018116100@njau.edu.cn (L.K.); 2018116099@njau.edu.cn (W.L.); 2020116098@stu.njau.edu.cn (T.Z.); zhuwenjiao@njau.edu.cn (W.Z.); qyang19@njau.edu.cn (Q.Y.); 2 Zhenjiang Institute of Agricultural Science of the Ning-Zhen Hilly District, Jurong 212400, China; jingdedao@163.com (D.J.); yujulong@126.com (J.Y.); shuzl2005@163.com (Z.S.); 3 College of Sciences, Nanjing Agricultural University, Nanjing 210095, China; lqhuangs@njau.edu.cn; 4 Center of Hydrogen Science, Shanghai Jiao Tong University, Shanghai 200240, China

**Keywords:** amylose, cadmium, field quality, hydrogen-based agriculture, hydrogen nanobubble water, rice

## Abstract

How to use environmentally friendly technology to enhance rice field and grain quality is a challenge for the scientific community. Here, we showed that the application of molecular hydrogen in the form of hydrogen nanobubble water could increase the length, width, and thickness of brown/rough rice and white rice, as well as 1000-grain weight, compared to the irrigation with ditch water. The above results were well matched with the transcriptional profiles of representative genes related to high yield, including up-regulation of *heterotrimeric G protein β-subunit gene* (*RGB1*) for cellular proliferation, *Grain size 5* (*GS5*) for grain width, *Small grain 1* (*SMG1*) for grain length and width, *Grain weight 8* (*GW8*) for grain width and weight, and down-regulation of negatively correlated gene *Grain size 3* (*GS3*) for grain length. Meanwhile, although total starch content in white rice is not altered by HNW, the content of amylose was decreased by 31.6%, which was parallel to the changes in the transcripts of the amylose metabolism genes. In particular, cadmium accumulation in white rice was significantly reduced, reaching 52% of the control group. This phenomenon was correlated well with the differential expression of transporter genes responsible for Cd entering plants, including down-regulated *Natural resistance-associated macrophage protein* (*Nramp5*), *Heavy metal transporting ATPase* (*HMA2* and *HMA3*), and *Iron-regulated transporters* (*IRT1*), and for decreasing Cd accumulation in grain, including down-regulated *Low cadmium* (*LCD*). This study clearly showed that the application of molecular hydrogen might be used as an effective approach to increase field and grain quality of rice.

## 1. Introduction

Rice is typically milled from brown rice to white rice, the most-often consumed form of rice, and more than 3 billion people use rice as their main food, particularly in Asian, South-American, and African countries [1]. Since it is rich in proteins, carbohydrates, vitamins, biologically active compounds, and organic acids, rice is generally good for human health [2]. However, rice normally appears to have more absorption of cadmium (Cd), a very toxic heavy metal caused by soil contamination and acidification, compared to other major cereal crops. This could result in the accumulation of Cd in rice grains exceeding the maximum permissible limit [3]. Meanwhile, amylose content is a key determinant of eating quality of rice [4]. Therefore, avoiding excessive Cd accumulation in white rice and improving field and grain quality, especially breeding or producing low-amylose content rice, are not only important for consideration during rice production, but also a challenge for scientific community.

The improvement of rice yield and quality normally utilizes molecular genetic selection combined with hybridization, which is mainly dependent on various rice germplasm resources [5]. The improved germplasm is normally integrated with the application of chemical fertilizers and pesticides [6]. During the last 25 years, the usage of recombinant genetic methods has been academically confirmed to be more efficient and reliable [7,8,9], but this technology requires relatively difficult approval protocols and has faced reluctance from consumers. The use of excessive pesticides and fertilizers in fields is another problem, since these can easily cause serious environmental pollution [6].

Molecular hydrogen is generally applied in clean energy. Although hydrogen gas (H_2_) is normally considered as a biologically inert gas, previous studies discovered that this gas could act as a therapeutic antioxidant in medicine [10]. Scientists have gradually realized that molecular hydrogen has anti-inflammatory, antioxidant, and anti-apoptotic effects in animal models and basic clinical research [11]. During the recent decade, the physiological functions of H_2_ in plants have been discovered, including enhancing plant tolerance against abiotic stress [12,13,14], and promoting plant growth and development [15]. For above cases, H_2_ might be integrated with other downstream gasotransmitters, including nitric oxide and hydrogen sulfide, as well as regulating some phytohormones [11,16]. In terms of postharvest storage period, previous results also revealed that H_2_ could prolong the shelf life of some vegetables, fruits, and flowers, including kiwifruit [17] and daylily bud [18], as well as lisianthus [19], carnation [20], and Chinese chive [21] when hydrogen-rich water, H_2_ fumigation, or magnesium hydride (a H_2_-releasing material) were separately applied. Since there were numerous functions of H_2_ observed in the vegetative growth stage and postharvest period, it is reasonable to deduce that molecular hydrogen might have significant influence in reproductive growth and seed developmental stages, both of which are very important for crop production, especially for rice.

Hydrogen-rich water (HRW) or saline is a typical form of molecular hydrogen used in plants and medicine, due to its feasibility and safety in laboratory [14]. While the low solubility and short residence time of H_2_ limit its wide application in practice, especially in paddy field, previous results have revealed that nano-bubble technology could be applied in aquaculture [22] and environment [23], since this approach could increase the content of targeted gas in water, prolong the time of the gas remaining in liquid, and improve the utilization efficiency of targeted gas. A recent study further discovered that the hydrogen nanobubble water (HNW) could alleviate copper toxicity in *Daphnia magna* since it could increase the solubility and the residence time of H_2_ in water, therefore efficiently enhancing antioxidant capacity [24]. For above reasons, HNW was used in our field experimental condition. 

The objective of this work was to investigate whether and how HNW could influence field and grain quality traits of rice, compared to the ditch water treated control group. Related results further supported the idea that the field trait, the qualitative characters, and quality of polished white rice were ubiquitously improved by HNW. Importantly, amylose content and Cd accumulation in white rice were significantly reduced. The related molecular mechanisms were preliminarily evaluated in terms of the transcript profiles of molecular marker genes.

## 2. Results

### 2.1. Seed Morphology of Rice Crops Irrigated with HNW

**Figure 1:**
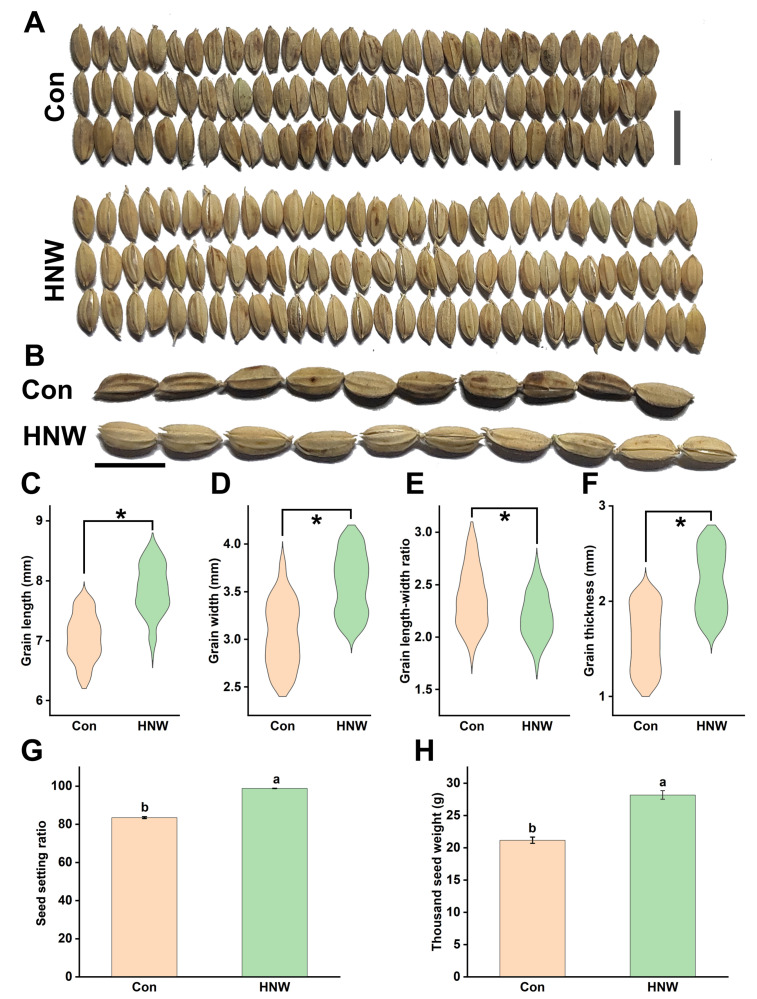
HNW positively influences the size and weight of grains. The photographs of representative 30 grains (**A**) and 10 grains (**B**) were taken (bar = 1 cm). Parameters of rice size, including grain length (**C**), width (**D**), length-width ratio (**E**), thickness (**F**), seed setting ratio (**G**), and thousand seed weight (**H**), were also provided. Asterisk indicates a significant difference between Con and HNW (n ≥ 1000, *p* < 0.001, two-way Student’s *t*-test). Data are mean ± SD (n = 3). Bars with different letters were significantly different in comparison with Con at *p* < 0.05.

The seed size of crops is an important trait of yield. The size of the grain influences many aspects of plant growth and development. In the trail experiment, we observed that the difference of length and width (in particularly) of rice grains (Figure 1A–D) harvested from plots either irrigated with HNW or ditch water (control) was clearly distinct. For example, the average length and width of the grains in HNW-treated group was about 11.4% and 15.1% greater than those from the control sample. Compared to grains harvested from the control, grain length-width ratio after irrigation with HNW was decreased by 4.3% (Figure 1E). Particularly, grain thickness showed pronounced improvement by 37.5% after HNW treatment (Figure 1F). The obvious increase in seed setting ratio was observed as well (Figure 1G). These results could be used to explain the significant increase in thousand-grain weight (about 23.8%; Figure 1H). 

**Figure 2:**
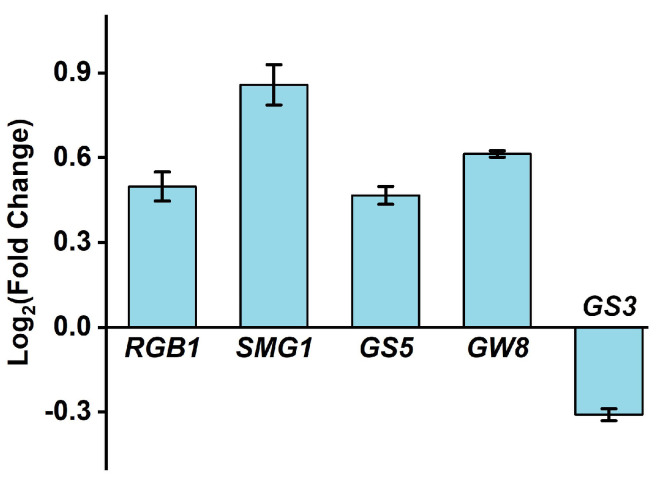
The results of gene expression in (young panicles) at the filling stage (5 September 2020) show that HNW may increase rice seed size. Then, transcripts of *RGB1*, *SMG1*, *GS5*, *GW8*, and *GS3* were analyzed by qPCR. Data are mean ± SD (n = 3).

**Figure 3:**
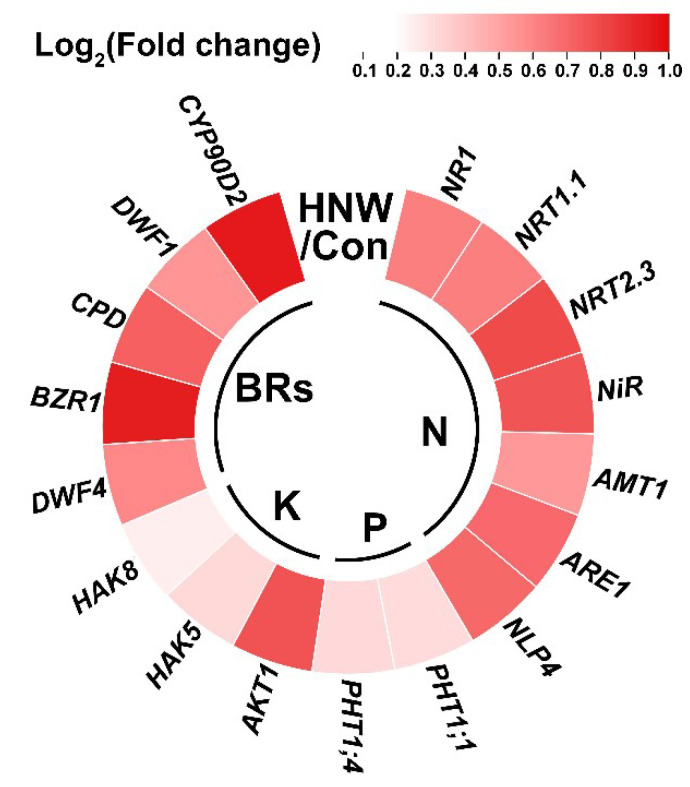
Molecular evidence showing that the results of rice seed size might be causally related to absorption of N, P, and K, as well as BRs signaling. Transcripts of genes related to absorption of N, P, and K in root tissues, and BRs signaling in young panicles were analyzed by qPCR. The samples were obtained on 5 September 2020. The different color means different log_2_(fold change) of HNW contrast Con.

Molecular evidence revealed that the above changes achieved by HNW were well matched with the transcriptional profiles of representative molecular markers responsible for high yield in rice young panicles (Figure 2). These included up-regulated *heterotrimeric G protein β-subunit gene* (*RGB1*) for cellular proliferation [25], *Small grain 1* (*SMG1*) for grain length and width [26], *Grain size 5* (*GS5*) for grain width [27,28], and *Grain weight 8* (*GW8*) for grain weight [29], after being irrigated with HNW. Meanwhile, the down-regulation of *Grain size 3* (*GS3*), negatively correlated with grain length [29], was observed after irrigation with HNW. Further results illustrated that the transcripts of representative genes related to the absorption of nitrogen (N), phosphorus (P), and potassium (K) in rice plants [30], including controlling N assimilation and transport, as well as P and K absorption, especially *Nitrate transporters 2.3* (*NRT2.3*), *Nitrite reductase* (*NiR*), *ABC1 repressor 1* (*ARE1*), *Nin-like protein 4* (*NLP4*), and *Potassium transporter 1* (*AKT1*), were obviously increased in HNW-irrigated rice roots (Figure 3). 

Since some of these genes, including *SMG1*, *GS5*, *NR1*, and *AMT1*, could be modulated by brassinolide (BRs) [26,27,31,32,33], so expression of specific genes related to BRs metabolism and signaling was further analyzed. As expected, the similar inducting profiles in *DWARF4* (*DWF4*), *Brassinosteroid signaling positive regulator* (*BZR1*), *Constitutive photomorphogenic dwarf* (*CPD*), *DWARF1* (*DWF1*), and *Cytochrome P450 90D2* (*CYP90D2*) mRNAs were observed in the HNW-treated rice young panicles (Figure 3). These changes might be used to explain the improvement of field and grain quantity in the HNW-irrigated group.

### 2.2. The Morphology of White Rice 

**Figure 4:**
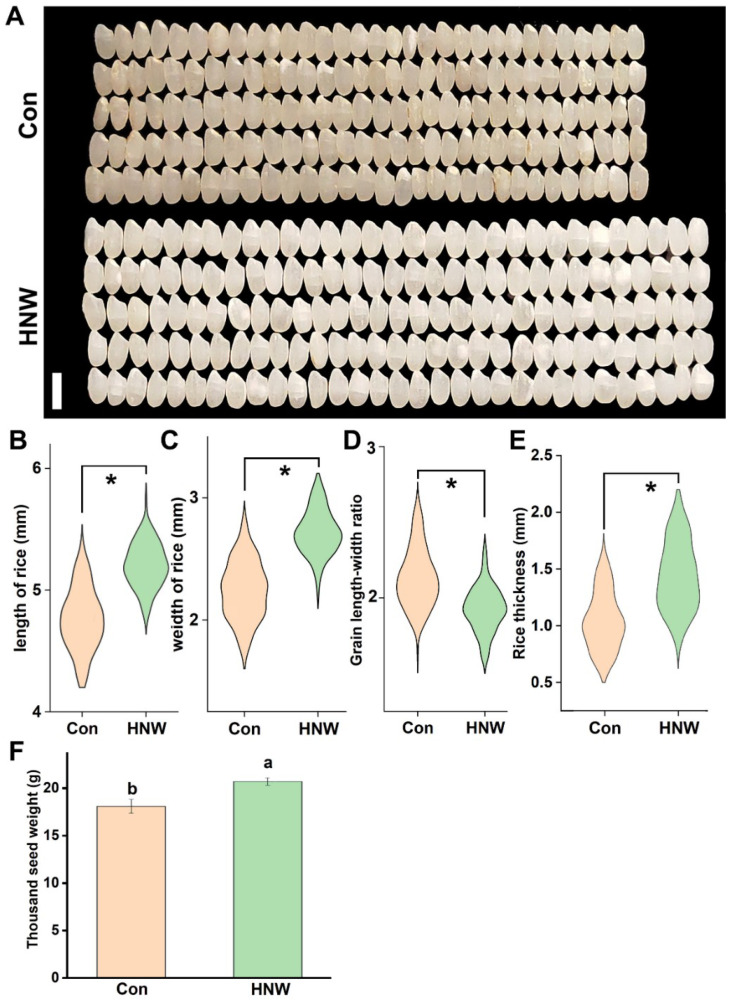
HNW positively influences the size and weight of white rice. The photographs of 30 white rice (**A**) were taken (bar = 0.5 cm). Parameters of white rice size, including length (**B**), width (**C**), length-width ratio (**D**), thickness (**E**), and thousand grain weight (**F**), were analyzed. Asterisk indicates a significant difference between Con and HNW (n ≥ 1000, *p* < 0.001, two-way Student’s *t*-test). Data are mean ± SD (n = 3). Bars with different letters were significantly different in comparison with Con at *p* < 0.05.

After processes, the rice grains are polished to edible white rice. Similar to the changes in grains (Figure 1), the length and width (especially) of white rice were obviously increased after HNW irrigation, compared to those of the ditch water-irrigated control group (Figure 4A–C). Although the changes in length-width ratio of white rice was negatively influenced by HNW (Figure 4D), both the thickness (Figure 4E) and thousand-seed weight (Figure 4F) displayed distinct improvements in comparison with the control group. Together, we found that molecular hydrogen could increase quantitative and qualitative traits of rice grain in field trials.

### 2.3. Qualitative Characters of White Rice Irrigated with HNW

**Figure 5:**
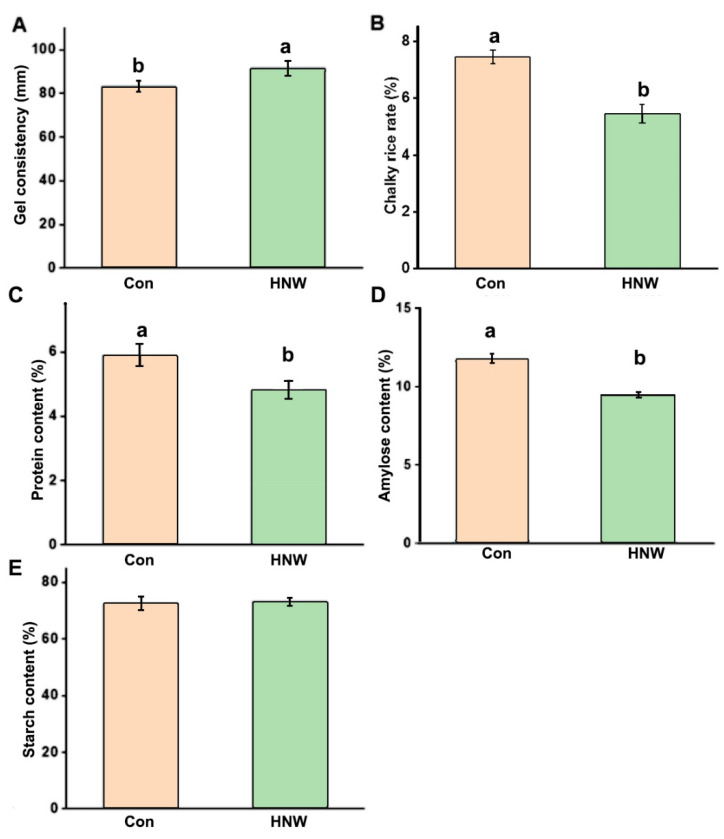
HNW positively influences qualitative characters of white rice. The gel consistency (**A**), chalky rice rate (**B**), contents of total protein (**C**), amylose (**D**), and starch (**E**) were analyzed. Data are mean ± SD (n = 3). Bars with different letters were significantly different in comparison with Con at *p* < 0.05.

Ample evidence found that the qualitative characters of white rice were closely related to gel consistency, chalkiness rice rate, contents of protein, total starch, and amylose contents [4]. Subsequent results discovered that compare to the control group (83.1 ± 2.4 mm), HNW could increase the gel consistency to 91.4 ± 3.4 mm (Figure 5A), a relatively perfect support for higher quality of eaten rice [34]. Strikingly, the chalkiness rate of rice was obviously decreased by HNW (Figure 5B). 

**Figure 6:**
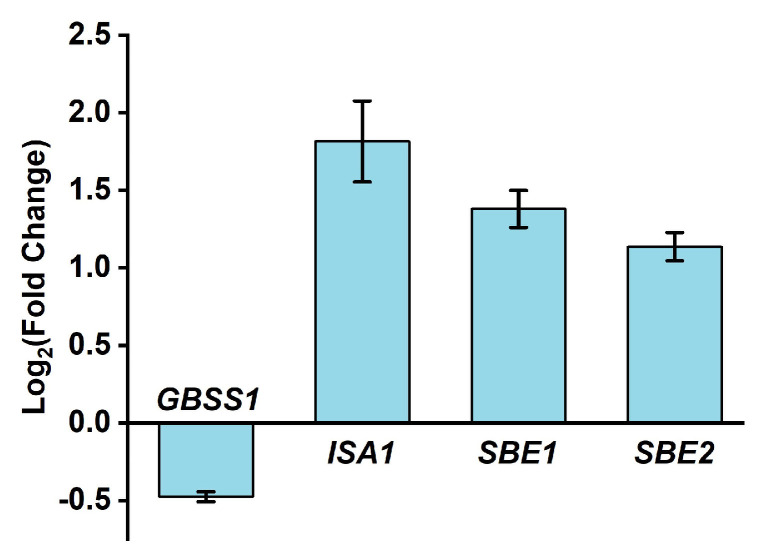
Changes of gene expression in leaves at the filling stage (5 September 2020) showing that HNW might decrease amylose accumulation. Transcripts of *GBSS1*, *ISA1*, *SBE1*, and *SBE2* were analyzed by qPCR. Data are mean ± SD (n = 3).

Subsequent results revealed that HNW irrigation could significantly reduce the total protein level (decreased by 19.8%; Figure 5C) and amylose content (decreased by 31.6%; Figure 5D) without altering the total starch content (Figure 5E). These are interesting findings. To probe the mechanism, some related genes controlling low amylose content in white rice, including *granule-bound starch synthase1* (*GBSS1*), *starch isomerase1* (*ISA1*), and *starch branching enzyme1*/*2* (*SBE1*/*2*), were analyzed (Figure 6). As anticipated, the changes in transcriptional expression of target genes related to amylose were well matched with the reduction in amylose content. These results clearly illustrated that HNW could decrease amylose synthesis.

**Figure 7:**
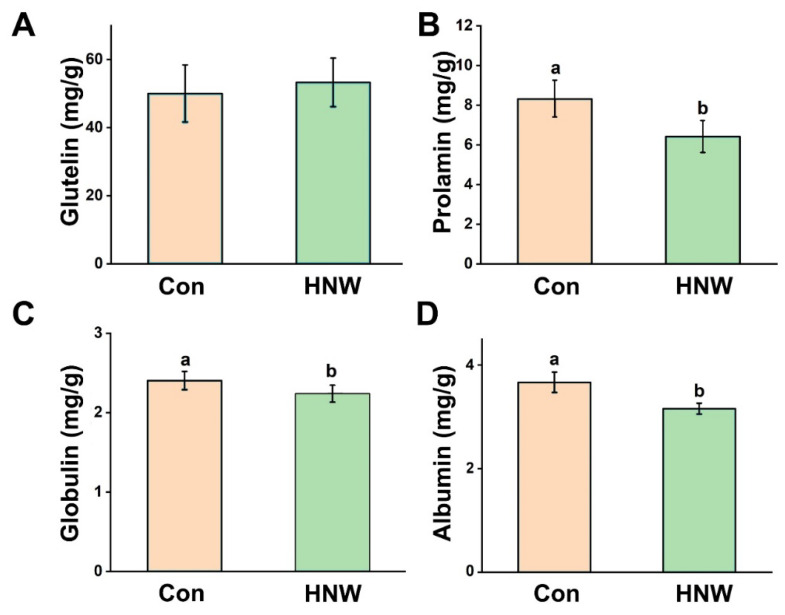
Composition of the crude protein in white rice. The contents of glutelin (**A**), prolamin (**B**), globulin (**C**), and albumin (**D**) were measured, respectively. Data are mean ± SD (n = 3). Bars with different letters were significantly different in comparison with Con at *p* < 0.05.

Although our results discovered that HNW could reduce total protein content in white rice (Figure 5C), the content of glutelin, an important and major storage protein in the endosperm of rice, was not significantly altered in response to HNW, with respect to the control sample (Figure 7A). Contents of other grain storage proteins, including prolamin (especially), globulin, and albumin (Figure 7B–D), were obviously impaired by HNW irrigation. Moreover, no significant differences were observed in the contents of the vitamin B1 and B5 in white rice after being irrigated with HNW or in the control group (data are not shown).

### 2.4. HNW Influences Contents of the Metal Ions in White Rice

**Figure 8:**
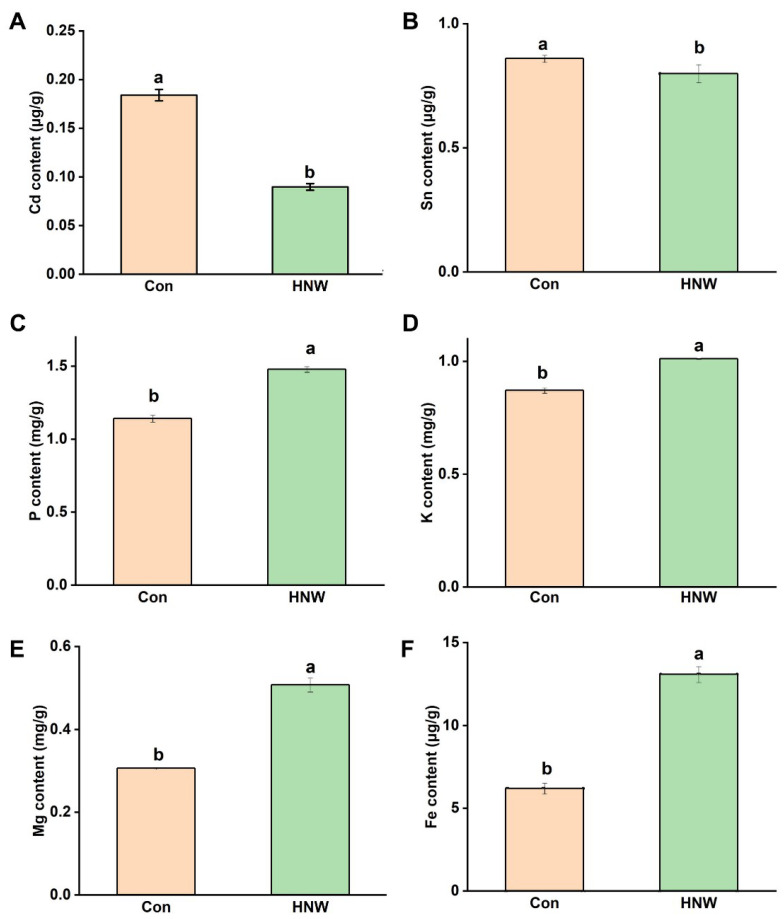
HNW affects the absorption of heavy metal and some nutrient element in white rice. The heavy metal content was analyzed, including Cd (**A**) and Sn (**B**). Meanwhile some nutrient elements, including phosphorus (**C**), potassium (**D**), magnesium (**E**), and iron (**F**), in white rice were further determined. Data are mean ± SD (n = 3). Bars with different letters were significantly different in comparison with Con at *p* < 0.05.

Further experiment was carried out to assess the possible link between element content of white rice and HNW irrigation. In our experimental conditions, the contents of Cd and tin (Sn) were reduced significantly by HNW, compared to those in control group (Figure 8A,B). The reduction in Cd accumulation by about 52% in white rice was very interesting since no significant difference in Cd content was discovered in soils sampled from the control and HNW-irrigated fields (data are not shown). Comparatively, we also noticed that HNW could increase the contents of some nutrient elements that are beneficial for both plants and humans, including phosphorus (P; Figure 8C), potassium (K; Figure 8D), magnesium (Mg; Figure 8E), and iron (Fe; Figure 8F) in white rice. 

**Figure 9:**
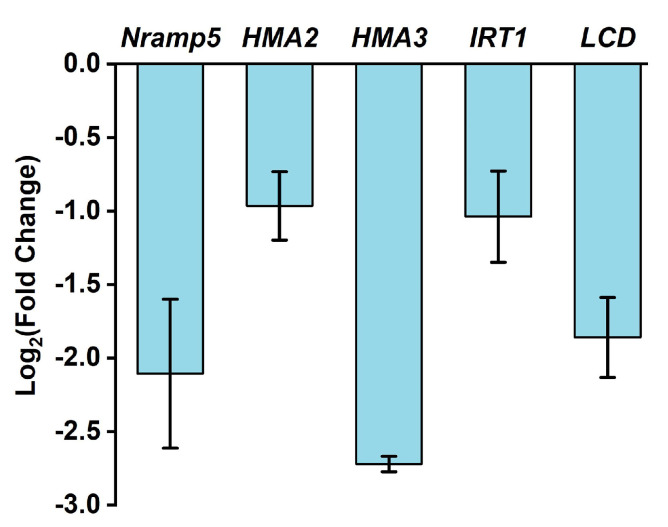
The results of gene expression in roots at the filling stage (5 September 2020) showing that HNW could decrease cadmium accumulation. Transcripts of *Nramp5*, *HMA2*, *HMA3*, *IRT*, and *LCD* were analyzed by qPCR. Data are mean ± SD (n = 3).

To gain insight into the molecular mechanism, the transcriptional abundance of genes responsible for reducing Cd content was analyzed in root tissues during the filling stage (Figure 9), and the change in gene expression could at least partly explain the variation of Cd content. For instance, *Natural resistance-associated macrophage protein* (*Nramp5*) [35], *Heavy metal transporting ATPase* (*HMA2 and HMA3*) [3,35], *Iron-regulated transporters* (*IRT1*) [3], and *Low cadmium* (*LCD*) [36], all of which were responsible for governing the entry and accumulation of Cd in plants, were remarkably down-regulated by HNW irrigation. Consistent with the changes in Cd content of white rice with or without HNW irrigation (Figure 8A), these molecular evidences clearly supported the idea that HNW irrigation was closely associated with the reduction of Cd accumulation via modulating transcriptional regulation.

## 3. Discussion

The presence of molecular hydrogen in plants has long been discovered [37,38,39]. In 2003, Dong and his colleagues discovered that exposing soils to hydrogen gas not only enhanced soil fertility, but also promoted plant growth of legumes and non-leguminous crops in both greenhouse and field trials. Afterwards, H_2_, at first glance a simple molecule consisting of only two hydrogen atoms, was progressively suggested to be “a global player” in plant physiology [12,13,40], especially in laboratory levels [11,16]. 

For rice, previous greenhouse experiments illustrated that the application of metallic magnesium-produced HRW could influence the reproductive fitness of rice in greenhouse experiments, and in particular, significantly inhibited the thousand seed weight of conventional rice and Bt-transgenic rice [41]. Pot-based experiments further showed that conventional electrolytically produced HRW could enhance rice tolerance against salinity [42] and heavy metal stress (Cd and lead) [43]. However, the direct evidence of H_2_ functioning in rice field trials, especially the influence in the field and grain traits, is still lacking. In this report, combined with nano-bubble technology, we provided physiological and molecular evidence for a previously uncharacterized role for H_2_ positive control of rice field and grain quality traits in a trial experiment. Our results are significant for both fundamental and applied plant biology. The above conclusion was based on the following evidence. 

First, the seed size of rice was an important issue in developmental biology, and was also an important part of seed yield [44,45]. A field study discovered the significant promotion of crop yield in spring wheat and barley when they were grown in H_2_-treated soil [46]. Consistently, our results clearly showed that unlike the changes in length-width ratio (Figure 1E and Figure 4D), the length, width, thickness, and thousand-seed weight of rice grain (Figure 1) and white rice (Figure 4) were remarkably improved by irrigating with HNW. The above results also suggested that compared to the length, the changes in the width of grain and the white rice are more sensitive to H_2_. 

These findings are inconsistent with those reported by Liu et al., in which they discovered the remarkable reduction in rice seed size in response to metallic magnesium-produced HRW [41]. The discrepancies may be attributed to different preparation methods for the hydrogen-based solution. Importantly, the possible negative influence achieved by other magnesium metabolites and/or possible pH alteration in the metallic magnesium-produced HRW could not be easily ruled out. A similar disadvantage was recently reported when magnesium hydride (MgH_2_) was used as a preservative for prolonging the vase life of cut flowers [22]. 

The results of qPCR analysis (Figure 2) further indicated that the abovementioned HNW governing seed size might be achieved by regulating the expression level of specific genes in young panicles during the filling stage that controls rice seed size [47]. It is well documented that the final size of rice grains is coordinately controlled by cell proliferation and cell expansion in the spikelet hull [48,49]. Interestingly, the transcript levels of several typical genes controlling seed size was remarkably modulated by H_2_. These include up-regulation of *RGB1* responsible for cellular proliferation [25], *SMG1* for grain length and width [26], *GS5* for grain width [27], and *GW8* for grain width and weight [29]. Previous results revealed that *SMG1*, which encodes mitogen-activated protein kinase kinase 4 (OsMKK4), could influence the seed size via influencing BR responses and the expression of BR-related genes [26]. *GS5* could keep BZR1/BRI1-associated receptor kinase1-7 (OsBAK1-7) on the cell surface, where it could interact with BZR1/BRI1 and enhance BR signaling, thereby affecting the grain size [27]. Meanwhile, the down-regulation of negatively correlated genes *GS3* for grain length and weight [29] were also consistent with the increased rice size in HNW-irrigated group. 

Ample evidence shows that genes controlling hormone levels (BRs, etc.) could be used to increase grain yields in rice [49]. For instance, seeds of the BRs receptor and metabolism mutant were smaller than wild-type [27]. By contrast, seeds of transgenic Arabidopsis lines overexpressing BRs synthetic genes were larger than wild-type seeds [50]. Previous studies showed that H_2_ might affect the synthesis and signaling of phytohormones, including auxin, abscisic acid, gibberellin, and ethylene [11,16]. Here, we observed that the transcripts of genes responsible for BRs synthesis/signaling (especially *CYP90D2*, *BZR1*, and *CPD*; Figure 3) and BRs-dependent seed size (*SMG1* [26]; *GS5* [31]; *NR1* [36]; and *AMT1* [33]) could be modulated by HNW (Figure 2 and Figure 3), all of which were consistent with the increased seed size of rice observed after harvesting and processing (Figure 1 and Figure 4). Thus, a cause-effect relationship between H_2_ and BRs governing rice grain size should be carefully investigated in the near future. 

It is well known that N, P, and K are principal nutrients that control crop productivity [51,52]. Therefore, the absorption and utilization efficiency of these nutrient elements are very important for rice yield [49,52]. To better understand the effects of HNW on crop production, the levels in P and K contents were evaluated. As expected, the irrigation with HNW could remarkably increase the accumulation of two elements in white rice (Figure 8C,D). Similar results were found in the positive changes in Mg and Fe ions (Figure 8E,F), both of which might be beneficial for human health in poor families, especially where rice is a staple food [53]. The reported results were also parallel to the transcriptional profiles of N, P, and K assimilation or the transport related gene (Figure 3), especially changes in *NRT2.3*, *NiR*, *ARE1*, *NLP4*, and *AKT1* transcripts, indicating that HNW control seed growth might be mediated by enhancing the assimilation of nutrient elements.

With the development of agriculture and food science and technology, the production for food crops also requires that agricultural products could improve qualitative characters and nutrient values [54]. Ample evidence confirmed that the increased gel consistency as well as decreasing protein and amylose contents in rice contributed greatly to the eating quality [4,54]. Our subsequent results shown in Figure 5 hinted that the white rice after irrigating with HNW had a better qualitative character, in terms of the changes in the above parameters, especially decreased amylose content. The latter change was supported by the transcriptional profiles of genes control of low amylose content (Figure 6). 

Human health is inseparable from delicious, nutrient-rich, and, especially, safe foods [55]. Rice is a plant that might accumulate Cd in the grain. The production of rice around the world is challenged by Cd pollution and the subsequent elevation of grain Cd levels [56]. Previous hydroponic experiments revealed that HRW could reduce the absorption and accumulation of Cd in alfalfa seedlings [28,57]. Here, the results of our field trials showed that the application with HNW could remarkably decrease Cd accumulation by 52% in white rice (Figure 8A). This is a new finding, although recent results confirmed that HRW could respectively decease Cd and lead (Pb) contents in shoot or root tissues of rice (Xiangyaxiangzhan cultivar; [43]). These findings also suggest that HNW irrigation might not only help rice against Cd stress, but also reduce the accumulation of Cd in white rice and its processed products. The reduction in Cd content in white rice might be related to the changes in transcription levels of genes in roots at the filling stage related to metal transport (Figure 9). These include genes governing the entry of Cd in plants, such as *Nramp5* responsible for regulating the transport of Cd into the vascular bundle [35], *HMA2* and *HMA3* responsible for loading of Cd in the xylem as well as transporting Cd from the cytoplasm to the vacuole [3,35], *IRT1* for Cd entry to plants [3], and *LCD* responsible for regulating the phloem Cd transport [36]. We also noticed that although gene expression of *Nramp5* and *IRT1* (Figure 9) favors competition between Fe and Cd uptake [58], higher content of Fe and reduction in Cd accumulation in white rice were observed after HNW irrigation (Figure 8F). These results reflect the complexity of molecular hydrogen functions in plants growth and tolerance against heavy metals.

## 4. Materials and Methods

### 4.1. Plant Materials and Field Experiments

**Figure 10:**
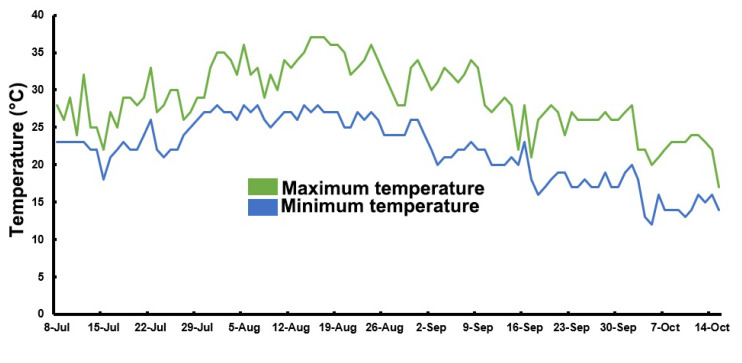
During rice planting, the field daily maximum and minimum temperature.

The rice (*Oryza sativa* L., Huruan1212 [59], a soft rice cultivar sensitive to sheath blight and rice blast; China Rice Data Center, https://www.ricedata.com, accessed on 9 October 2021) seeds were obtained from Zhenjiang Agricultural Research Institute, China. For further analysis, thirty-day-old rice seedlings were transplanted into paddy fields in Jurong, Jiangsu province, China (longitude 119.26° E and latitude 31.95° N), in early June 2020, and allowed to grow in natural conditions. There were two paddies used for HNW and ditch water (Con) treatment groups, and every paddy was about 150 m^2^ in this trial experiment. Additionally, the rice plants were grown without any chemical fertilizers and pesticides in the whole growth season. The daily highest and lowest temperature during the planting was recorded (Figure 10).

Rice plants were grown under the normal growing conditions until booting stage, and then HNW irrigation was imposed between 14 August 2020 and 15 October 2020, with one time per week, until the harvest stages. Each paddy field was irrigated with about 3 tons of water (HNW or ditch water) per time. 

The HNW was produced by a hydrogen nanobubble water generator (HIM-22, Guangdong Cavolo Health Technology, China). Generally, when preparing HNW by electrolysis system, the voltage used for electrolysis was higher than 4.5 volts, and the current was higher than 35 amperes. H_2_ produced from water electrolysis was infused into nanobubbles by a nanobubble aerator, and then dissolved into the ditch water. Before irrigating, the concentrations of dissolved H_2_ were determined by a portable dissolved hydrogen meter (ENH-2000, TRUSTLEX, Japan; calibrated by gas chromatography). In our experimental conditions, H_2_ content in the HNW was about 0.5 mM (1000 ppb). The half-time of dissolved H_2_ in the above HNW was at least 3 h. Additionally, the diameter of the nanobubbles of hydrogen gas in the HNW was about 60–550 nm (determined by the NS300, Malvern Panalytical, Britain). 

The rice was harvested on 1 November 2020, and the grains were then photographed and recorded. Afterwards, the grains were processed into white rice for further analysis. At least 1000 grains/white rice were randomly selected to record the sizes. Seed setting ratio and thousand seed weight were measured in triplicate. Each replicate included 1000 grains/white rice, and the total grains/white rice was 3000 (1000 × 3). Finally, about 15 g white rice was randomly selected and ground into powder for the further analysis. 

### 4.2. Determination of Qualitative Characters

The content of total protein was measured by an automatic kieldahl apparatus (KDN-08A; Hongji, Shanghai, China) according to the previous study [60].

The amylose contents were determined using a dual-wavelength iodine-binding method [61].

The determination of gel consistency, chalky rice rate, and starch content were carried out according to the previous method [4].

### 4.3. Determination of Ion Content

According to previous reports [62], ion contents in white rice and soil were detected by using a Digital Block Sample Digestion System (LabTech ED54 DigiBlock). The metal ions contents were determined by an Inductively Coupled Plasma-Optical Emission Spectrometer (ICP-OES, iCAP 7000, Thermo Fisher).

### 4.4. Determination of Albumin, Globulin, Prolamin, and Glutelin Content

After extraction [63], the composition of white rice protein, including albumin, globulin, prolamin, and glutelin, was determined by using BCA Protein Assay Kit (TaKaRa, Beijing, China).

### 4.5. Real-Time Quantitative Reverse Transcription-PCR (qPCR)

The leaves, young panicles, and roots were randomly collected from rice plants in the filling stage (20 September 2020), and further frozen in liquid nitrogen immediately. After the extraction of total RNA and the synthesis of cDNA, the quantitative real-time PCR (qPCR) was carried out. The primers’ sequences are shown in Supplementary Table S1. The relative expression levels of corresponding genes are presented as values relative to those of corresponding control samples, after normalization with two reference genes *OsActin1* and *OsUbi*. The results of relative genes expression levels were analyzed by the 2^−ΔΔC^_T_ method [64]. All determinations were carried out using three separate RNA samples, and each run in triplicate.

### 4.6. Statistical Analysis

All results are shown as the mean values ± SD of three independent experiments with three biological replicates for each. By using Origin 2021, the data were analyzed by Student’s *t*-test or one-way analysis of variance (ANOVA). *p* < 0.05 was considered statistically significant.

## 5. Conclusions

In conclusion, this study demonstrated that the application of HNW during the growth and development stage of rice could not only significantly increase the field and grain quantity of rice grains and white rice, but could also improve qualitative characters, maintain nutrition ingredients, and alleviate accumulation of Cd of the white rice. As we know, this is the first time using HNW in field trails, which might act as an important strategy for enhancing sustainable crop production. Importantly, results on the transcriptional profiling seem to give some answers on the question of how HNW increased field and grain quality traits. Since a multitude of different signaling pathways could be closely associated with molecular hydrogen functions in plants [16], the existence of a simple cause-and-effect chain seems very unlikely.

Compared to the direct usage of hydrogen gas in field soil [46], irrigation with HNW is a relatively convenient and cheaper approach; thus, this method might be used in large-scale agriculture. We also admitted that hydrogen-based agriculture is just at the beginning stage, and many field trials and deeper molecular mechanisms should be further carried out and elucidated.

## Data Availability

This statement if the study did not report any data.

## Supplementary Materials

The following are available online at https://www.mdpi.com/article/10.3390/plants10112331/s1, Table S1: The sequences of primers for qPCR, References [65,66,67,68,69,70,71,72,73,74,75] are cited in the Supplementary Materials.
