# Supplementary material for: Molecular Hydrogen Increases Quantitative and Qualitative Traits of Rice Grain in Field Trials
Source: Plants (Basel). 2021 Oct 28;10(11):2331. doi: 10.3390/plants10112331 (PMC8624507; doi:10.3390/plants10112331)
Supplement: Supplementary file 1 [file plants-10-02331-s001.zip › plants-1436192-supplementary.pdf]

Supplementary Table S1. The sequences of primers for qPCR

Gene name	Supply Reference	Forward primer	Reverse primer
Actin1	[26]	ACCATTGGTGCTGAGCGTTT	CGCAGCTTCCATTCCTATGAA
Ubi	[25]	TGAAGACCCTGACTGGGAAG	CACGGTTCAACAACATCCAG
RGB1	[25]	TGGTCTTTCTTCTGATGGGAGTG	CACAAGGAACTTACAACAACACAGG
SMG1	[26]	TGCTATGTACGTCGCCATCCAG	AATGAGTAACCACGCTCCGTCA
GS5	[26]	AGTGGACTGCTTCCAGGGAAG	CACGCAGTACCGAGAACTGA
GS3	[26]	GAACTCCTGATCCATTCATAACGATT	CAAACAGCGAAACTTCTTCAAGAA
GW8	[26]	AGGAGTTTGATGAGGCCAAG	GCGTGTAGTATGGGCTCTCC
NR1	[65]	CCAATTCTTTCATCGTGTTCT	CATGCAGCATTTCGTTTCT
NRT1.1	[66]	CAATTGGACCTATTTCGTAGCC	GCAGAAATGGTAAAACCCC
NRT2.3	[66]	CGCTGCTGCCGCTCATCCG	CCGTGCCCATGGCCAGAC
NiR	[66]	TGGCTCATCGACGAACTTGGAATG	TCCCTCCTCTGCCATTTCTTGTC
AMT1	[66]	AGCGAAGGAAGAAATCACG	CCAAACAGAAACTGGCAATC
ARE1	[67]	TGCTGTCATCCTTTCTCCAC	CTTGATACGTCTGAGCATCTCG

NLP4	[66]	AGTCGCCATTCTGACGAGAACT	TGGGTACTCCAGTTTAGTGGAG
PHT1;1	[68]	CATGTCGCTCGAGGTTATCTC	AGAGATGACACCAATGGTTAGC
PHT1;4	[68]	ATGGATCCAATTΓCAGCGACATTPTCTC	TACTGCAGTGGTACACTAGCAGAACCAGAA
AKT1	[69]	GCTTCCAAAGGAAACGAGCAA	GCAAGCGTATAAGCCCGTGTC
HAK5	[69]	CATTGTGGACTATTTTGAAAGAA	GGAGAACTACAGAAAAGCCAATC
HAK8	[69]	CATTGAAGGACTATTTGAA	AGAACTTACAGAAAGCCA
DWF4	[26]	TCGATGCTGGACATCCTGGG	CTGCCTTGAGAGCGTCAGAG
BZR1	[26]	CTTCACGGGGCTCCGGGCGCTCGGGAACTAC	GGAGGAACGGGAGGAGGCAGGAGGCGCTCG
CPD	[70]	TTACCGCAAAGCCATCCAA	TCATCACCACCACCGTCAAC
DWF1	[71]	ATGGCAGATCTGCAGGAGCC	TTACGCCTCATCAGCGTAGGC
CYP90D2	[70]	ATGGCGAGGCTGATACAGAGG	CTAGTCGTCGTCCTCCTTGGC
GBSS1	[72]	GATGCGTTTCAGCCTTCTTTG	AGTATGGGTTGTTGTTGAGGTTTAG
ISA1	[73]	GTGGCCTTCTCCACGAAAGA	CTCTGGGAGTCCAACAACCG
SBE1	[74]	TGGCCATGGAAGAGTTGGC	CAGAAGCAACTGCTCCACC
SBE2	[75]	ATGCTAGAGTTTGACCG	AGTGTGATGGATCCTGC

		C	
Nramp5	[35]	CAGCAGCAGTAAGAGCAAGATG	CAGCAGCAGTAAGAGCAAGATG
HMA2	[35]	GCAGATCAAGTCACCCCATGG	GCCATCACCAACCATCAGCGT
HMA3	[35]	TCCATCCAACCAAACCCGGAAA	TGCCAATGTCCTTCTGTTCCCA
IRT1	[58]	ATGAGGTCGGTGCTCGTCT	CGGGCTGTTGTCCCTGTA
LCD	[36]	CTATGATTTCATCGGATCTACCGACTG	CTAAGAACCAAAAACTCCTAACAGGAG

References

65. Yang, B.; Wang, X.M.; Ma, H.Y.; Li, Y.J.; Dai, C.C. Effects of the fungal endophyte Phomopsis liquidambari on nitrogen uptake and me-tabolism in rice. *Plant Growth Regul.* **2014**, *73*, 165–179.
66. Yan, M.; Fan, X.; Feng, H.; Miller, A.J.; Shen, Q.; Xu, G. Rice OsNAR2.1 interacts with OsNRT2.1, OsNRT2.2 and OsNRT2.3a nitrate transporters to provide uptake over high and low concentration ranges. *Plant, Cell Environ.* **2011**, *34*, 1360–1372, doi:10.1111/j.1365-3040.2011.02335.x.
67. Wang, Q.; Su, Q.; Nian, J.; Zhang, J.; Guo, M.; Dong, G.; Hu, J.; Wang, R.; Wei, C.; Li, G.; et al. The Ghd7 transcription factor represses ARE1 expression to enhance nitrogen utilization and grain yield in rice. *Mol. Plant* **2021**, *14*, 1012–1023, doi:10.1016/j.molp.2021.04.012.
68. Ye, Y.; Li, P.; Xu, T.; Zeng, L.; Cheng, D.; Yang, M.; Luo, J.; Lian, X. OsPT4 Contributes to Arsenate Uptake and Transport in Rice. *Front. Plant Sci.* **2017**, *8*, 2197, doi:10.3389/fpls.2017.02197.
69. Okada, T.; Nakayama, H.; Shinmyo, A.; Yoshida, K. Expression of OsHAK genes encoding potassium ion transporters in rice. *Plant Biotechnol.* **2008**, *25*, 241–245, doi:10.5511/plantbiotechnology.25.241.
70. Sakamoto, T.; Matsuoka, M. Characterization of CONSTITUTIVE PHOTOMORPHOGENESIS AND DWARFISM Homologs in Rice (Oryza sativa L.). *J. Plant Growth Regul.* **2006**, *25*, 245–251, doi:10.1007/s00344-006-0041-6.
71. Tsukagoshi, Y.; Suzuki, H.; Seki, H.; Muranaka, T.; Ohyama, K.; Fujimoto, Y. Ajuga Δ24-Sterol Reductase Catalyzes the Direct Reductive Conversion of 24-Methylenecholesterol to Campesterol. *J. Biol. Chem.* **2016**, *291*, 8189–8198, doi:10.1074/jbc.m115.703470.
72. Crofts, N.; Satoh, Y.; Miura, S.; Hosaka, Y.; Abe, M.; Fujita, N. Active-type starch synthase (SS) IIa from indica rice partially com-plements the sugary-1 phenotype in japonica rice endosperm. *Plant Mol. Biol.* **2021**, doi:10.1007/s11103-021-01161-9.
73. Utsumi, Y.; Nakamura, Y. Structural and enzymatic characterization of the isoamylase1 homo-oligomer and the isoamylase1–isoamylase2 hetero-oligomer from rice endosperm. *Planta* **2006**, *225*, 75–87, doi:10.1007/s00425-006-0331-z.
74. Satoh, H.; Nishi, A.; Yamashita, K.; Takemoto, Y.; Tanaka, Y.; Hosaka, Y.;Sakurai, A.; Fujita, N.; Nakamura, Y. Starch-branching enzyme I-deficient mutation spe-cifically affects the structure and properties of starch in rice endosperm. *Plant Physiol.* **2003**, *133*, 1111–1121.

75. Nakamura, Y.; Utsumi, Y.; Sawada, T.; Aihara, S.; Utsumi, C.; Yoshida, M.; Kitamura, S. Characterization of the reactions of starch branching enzymes from rice endosperm. *Plant Cell Physiol.* **2010**, *51*, 776–794, doi:10.1093/pcp/pcq035.
